# Assessing Sustainable Development of Deep Aquifers

**DOI:** 10.1007/s11269-023-03529-6

**Published:** 2023-05-16

**Authors:** Annette Dietmaier, Thomas Baumann

**Affiliations:** TUM School of Engineering and Design, Chair of Hydrogeology, Technical University Munich, Arcisstr. 21, Munich, 80333 Bavaria Germany

**Keywords:** Sustainable exploitation, Cluster analysis, Deep groundwater, Early warning system

## Abstract

Deep groundwater aquifers are exploited for a variety of purposes. In general, impermeable rock layers protect these aquifers from anthropogenic influences. As such, they are a last resort for groundwater in a pre-industrial state, and a crucial resource in cases of emergency, such as floods contaminating shallow groundwater. The EU Water Framework Directive (WFD) provides the regulatory framework to protect its quality and quantity. Recent monitoring of the hydrochemical state of Upper Jurassic wells in Bavaria and Austria has shown fluctuations that were connected to new exploitation activities and might indicate an unsustainable development of the aquifer. We propose a new workflow in accordance with the WFD which uses clustering algorithms to assess these fluctuations. Our data consists of 5 to 42 hydrochemical analyses per well with yearly sampling intervals spanning up to 30 years. From the cluster analysis we derived thresholds for two corridors: Natural Range Corridor (NC) and Action Corridor (AC). While the NC represents a well-specific natural variation range, the AC hints towards unsustainable development and should trigger a detailed (re)assessment. To show the potential of the new method, the workflow was applied to two wells with different geological characteristics. Distinct fluctuation events were clearly recognized and can be used in the context of an early warning system, such that malign hydrochemical variations can be detected before they become legally problematic to well operators. Our workflow thus provides a novel, robust, and reproducible method to assess the grade of sustainability at which a well is exploited and ensures a good status of a unique and important resource.

**Supplementary Information:**

The online version contains supplementary material available at 10.1007/s11269-023-03529-6.

## Introduction

Deep groundwater is protected from anthropogenic influences by hundreds of meters of rock matrix with limited permeability and high retardation potential. Under this premise, deep groundwater reservoirs constitute an extraordinary source of clean water, especially during times in which more shallow and surface water bodies are contaminated or depleted. The importance and wide range of applications of this resource results in conflicts over concurrent exploitation, e.g. large net discharge for technical purposes, geothermal energy, and drinking water production (Wycisk et al. 2003; Goldscheider 2005; Panagos et al. 2013; Baiocchi et al. 2013).

An aquifer is considered under stress if the cumulative withdrawal rate exceeds 20 % of the annual recharge rate (Arle et al. 2017). This value primarily ensures quantitative sustainability. However, groundwater flow velocities in deep aquifers are usually very slow. Thus, any withdrawal will change the age structure of the water body as more recent water replaces the water withdrawn. Analyzing age structures among bottled waters and evidence of pesticides present in these waters, Baumann (2013) shows that the exploitation of deep groundwater can change the age of the produced water from more than 1000 years to less than 30 years. Thus, on human time scales, deep groundwater bodies should be considered as a non-renewable resource (Ungemach et al. 2005). Nevertheless, aquifer-specific sustainable management plans currently do not exist for many deep aquifers.

Using groundwater age as a direct input parameter to assess the impacts of local exploitation schemes might seem intuitive. However, current age determination methods exhibit a range of uncertainties and therefore a lack of sensitivity when assessing small changes in the age structure, making groundwater age a questionable indicator for groundwater sustainability (Ferguson et al. 2020). Instead, we propose to use the hydrochemical signatures of individual wells and their changes as a sensitive indicator for (non-)sustainable well development. This premise assumes that over-exploitation of aquifers results in hydrochemical changes (Li et al. 2013).

The European Water Framework Directive (WFD; European Parliament and Council (2000)) sets the legal context for the assessment of groundwater bodies. It states that a “good quantity and quality status” must be reached for all specified groundwater bodies until 2027 (Foster and Custodio 2019). It also defines the good chemical status using electric conductivity (EC) to examine the effects of saline or other intrusions, and emphasizes anthropogenic influences and pollutants (European Parliament and Council 2000). These guidelines have had positive impacts on shallow groundwater bodies vulnerable to anthropogenic activities (Foster and Custodio 2019). However, deep aquifers underlie vastly different stressors and are less affected by anthropogenic influences, given their location of 150 m to 7000 m underground (Kang et al. 2019). Deep groundwater aquifers, such as the Upper Jurassic of the North Alpine Foreland Basin (NAFB), are at a small risk of receiving pollutants directly from anthropogenic sources. However, they may assume a “bad” chemical state (according to the WFD) e.g. through the intrusion of oil, gas or saline waters from higher or lower strata (European Parliament and Council 2000; Kang et al. 2019).

Examples of national industry standards implementing the WFD’s core ideas are the EU mineral water directive (European Parliament and Council 2000) or the German Spa Association’s (GSA) *“Definitions and quality standards for the nomination of health resorts, resort towns and curative sources”* (Deutscher Heilbäderverband and Deutscher Tourismusverband 2016). There are other national equivalents of WFD implementations with similar shortcomings. The characteristics discussed here are thus not limited to this German legal framework but can be extrapolated internationally. The GSA framework denotes threshold values for ingredients with balneo-therapeutical use, and allows

a ± 20 % and ± 50 % variation in the concentrations of characteristic ingredients and of ingredients with a concentration of< 20 mg/L, respectively. However, these thresholds are detached from a well’s natural variability. Hence, fluctuation characteristics which are clearly not part of the well’s natural fluctuation, might go unnoticed in the mandatory yearly hydrochemical analysis, if they lie within the allowed corridors. Thus, the generic value of ± 20 % fails to detect unsustainable well developments.

Temporal variations in hydrochemical parameters indicate the response of a heterogeneous inflow regime to varying withdrawal rates. This might be caused by, among other factors, seasonal fluctuations in heating demand or in the number of guests in balneological treatments, unpredictable events such as lock-downs during the COVID-19 pandemic, or pump malfunctions. Furthermore, reinjection of cooled-off waters or lack thereof must be considered. Most yearly sampling campaigns are scheduled within a ± 2 - 3 week time window during the calendar year, hence the sampling takes place at a similar operational state of the well.

The paramount importance of maintaining the quantitative and qualitative integrity of deep groundwater aquifers stands in contrast to a lack of suitable monitoring and management solutions. The result of this contradiction is the need for sound and reproducible methods to assess malign changes of a well’s hydrochemical signature whilst determining its natural fluctuation range. These methods must also be robustly applicable to rudimentary data sets at unequal time intervals.

Determining the natural range must be achieved through clustering data points representing the well’s natural fluctuation. Clustering algorithms follow the premises of pattern recognition, grouping data points with similar characteristics, and subdividing large data sets into smaller clusters in an unbiased fashion (Fu et al. 1976; Kaufman and Rousseeuw 1990). Difficulties arise when applying clustering methods on sparse training data. In other words, one aims to group data into homogeneous clusters without using any information pertaining to the groups of the samples (Lee 1981). Thus, having good knowledge of the data structure and the purpose of clustering is imperative. Two clustering algorithms from opposite sides of the clustering algorithm spectrum are DIvisive ANAlysis (DIANA) and k-means. DIANA is a hierarchical (top-down) clustering approach. It splits the initial data set into two clusters defined by their Euclidian distance to the most different data points. The resulting clusters are split up until each remaining cluster contains only one single data point (Patnaik et al. 2016).

K-means analysis, an agglomerative, bottom-up algorithm, approaches the clustering process in the reverse order (Kaufman and Rousseeuw 1990; Patnaik et al. 2016). As one of the simplest unsupervised learning algorithms (Kodinariya and Makwana 2016), it partitions a data set into *k* groups, *k* being set by the user (Wagstaff et al. 2001). This requires the investigator to have good prior knowledge off the data’s structural characteristics. The locations of these *k* cluster centers are then iteratively refined using the Euclidian distance of the instances to the respective cluster center (Wagstaff et al. 2001).

Cluster analysis in combination with hydrogeochemical analyses have been established in examining spatial and temporal patterns of groundwater chemistry (Yang et al. 2020; Heine et al. 2021; Kim et al. 2003; Wang et al. 2015). Most studies, including recent investigations, utilizing this approach, focus on spatial hydrogeochemical zonation and use temporal averages representing the entire sampling period (Yang et al. 2020), neglecting temporal dynamics (Sayemuzzaman et al. 2018; Heine et al. 2021). Studies which focus on temporal analysis tend to apply cluster analysis to data of separate sampling periods in order to track changes between these periods over a larger geographic area, rather than at individual well sites (Pacheco Castro et al. 2018; Yang et al. 2020; Thyne et al. 2004; Hussain et al. 2008). Cluster analysis has also been used in groundwater monitoring programs. Ribeiro and Macedo (1995) employ a hierarchical cluster analysis (HCA) in order to establish groups of stations characterized by similar temporal patterns, after applying principal component analysis to the data set in order to define intercorrelation structures between the variables, and then analysing temporal variations of the resulting indices by the Mann-Kendall test. Daughney and Reeves (2006) apply HCA on the temporal trends in groundwater chemistry which they previously determined by using the Mann-Kendall test. They emphasize the importance of defining baseline rates of changes in groundwater quality at the national scale, since a given well “cannot be defined as ’affected’ or ’abnormal’ unless the threshold limit of normality (baseline) has been previously defined” (Daughney and Reeves, 2006). Considering the range of conditions groundwater may encounter in natural systems, e.g. aquifer lithology, confinement, recharge source and age, it is important to define baselines as ranges of values rather than as single numbers. In their study, they define these ranges using percentiles (5th, 25th, 75th and 95th (Daughney and Reeves 2006)). At the time of writing (April 2022), we are not aware of any attempts to utilize clustering analysis methods to define dynamic natural temporal ranges of water quality at individual wells, rather than on aquifer level or through arbitrary value ranges.

Thus, based on data gathered in the Lower Bavarian and Upper Austrian part of the NAFB, the primary research question for this study was to define a well’s natural state (considering its total concentration of main ions and trace substances), using reproducible statistical analysis methods and rudimentary data sets. Further, we test the functionality of the developed framework as an early warning system for changes in the well’s hydrochemistry.

This study offers a novel approach of determining the typical inherent fluctuation ranges at each individual well in the NAFB, rather than employing an arbitrary value. Knowing this characteristic fluctuation is at the heart of our understanding of how sustainable geothermal groundwater exploitation is and how competing well operators using the same aquifer can coexist sustainably.

## Methods

### Study Area and Data

**Fig. 1:**
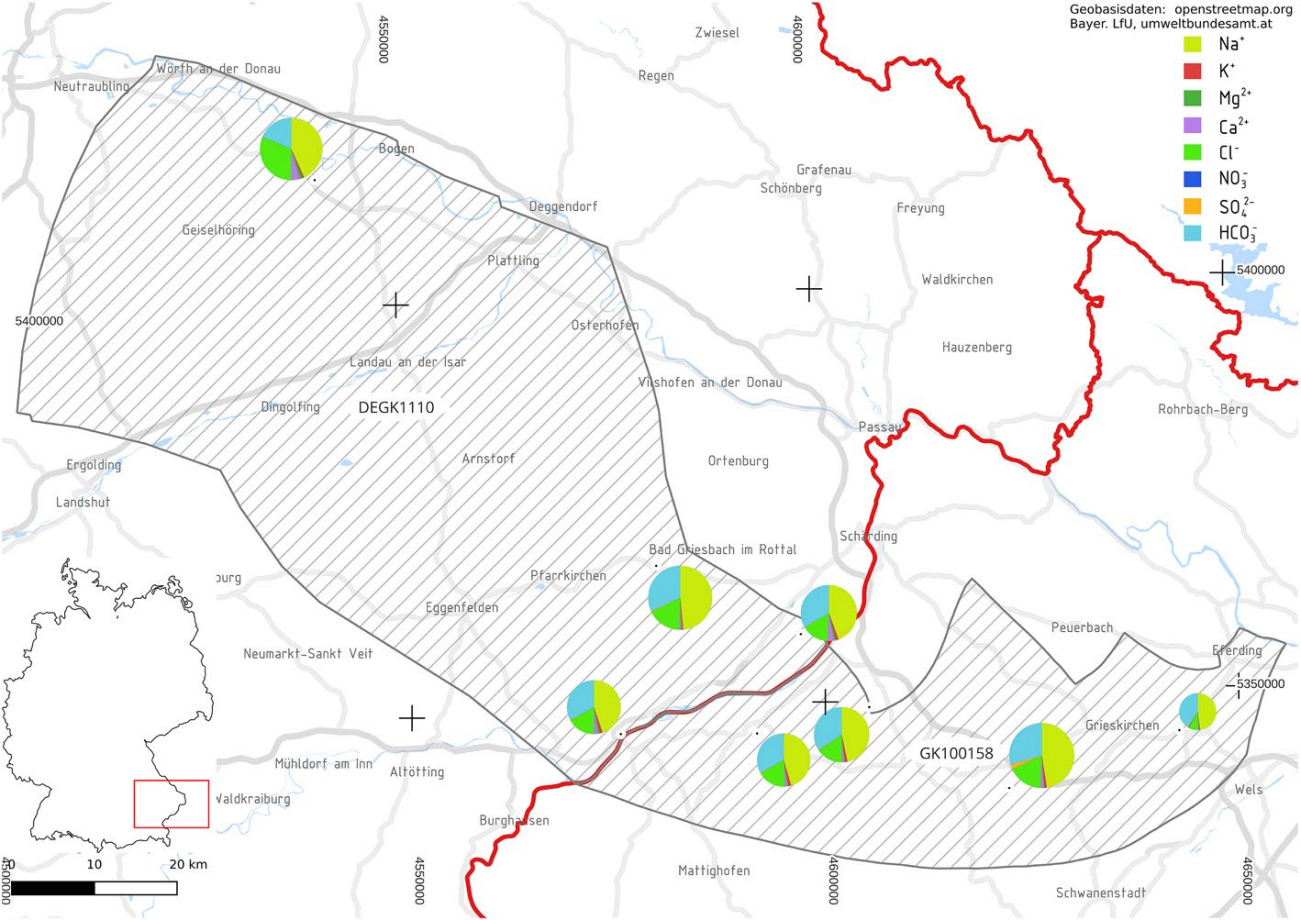
Study area: DEGK1110 in Lower Bavaria and GK100158 in Upper Austria. The size of the pie charts representing the hydrochemical characteristics of the examined wells indicates TDS

We selected 8 out of 22 geothermal wells (Fig. 1) exploiting the Upper Jurassic in the Northern Alpine Foreland Basin (NAFB) in Germany and Austria for analysis (Fig. 1). Previous studies (Mayrhofer et al. 2014; Heine et al. 2021; Birner et al. 2012) have shown that these carbonates are connected and constitute one deep groundwater body.

The selected wells produce hot water for balneological, district heating, and power generation purposes. The number of available analyses ranges from 5 to 42. While the earliest analysis was sampled in 1939 at BF1, only a few historic analyses were available. Yearly sampling started in the 1990s. Data include physical parameters (such as flow rate, electric conductivity (EC), temperature and pH) and hydrochemical parameters (main ions and trace ions relevant for balneotherapy). While most analyses at the German wells were performed by the Institute of Hydrochemistry at the Technical University of Munich, various other laboratories were involved. All data were stored in a PostgreSQL database and connected to QGIS through PostGIS for spatial representation (PostgreSQL Global Development Group (2021); PostGIS Project Steering Committee (PSC) (2021); QGIS Development Team (2021)).

### Descriptive Statistics

Descriptive statistics were calculated using R (R Core Team 2020) for total dissolved solids (TDS), main ions, ions with relevance for balneotherapy and physical parameters. For all following analyses, we define TDS as the sum of the eight main ions and characterizing trace ions (Na^+^, K^+^, Ca^2+^, Mg^2+^, F^-^, CI^-^, SO_4_^2-^ and HCO_3_^-^). An aggregated Schoeller diagram and two Piper diagrams (Online Resource 1) describe the wells’ general hydrochemical characters. In order to describe the hydrochemical signatures in more detail, we must consider the inflow pathways affecting hydrochemical processes in the rock matrix and thereby TDS and individual parameter concentrations.

### Cluster Analysis

This study employs two clustering algorithms: DIANA and k-means. By employing two methods from opposite ends of the clustering methods spectrum (bottom-up vs. top-down, unconditioned vs. preconditioned), we cover a broad array of approaches. Both methods were performed using the R packages “stats”, “factoextra” (Kassambara and Mundt 2020), “cluster” implementing methods developed by Kaufman and Rousseeuw (1990) and Maechler et al. (2021), and “gridExtra” (Auguie and Antonov 2017).

Aiming to discern data points characterized by low TDS, those with TDS values higher than usual, and a value range between these two extremes, we grouped the data sets into three clusters (*k* = 3) for the k-means analysis. DIANA presents its results in a dendrogram which displays the similarity between two clusters. The larger the vertical distance between two clusters, the more dissimilar they are to each other (Kaufman and Rousseeuw 1990). The number of clusters thus depends on the vertical height value defined as a cut-off. Thus, we determined an appropriate height value for each well.

We ran both clustering algorithms on data without prior normalization because the absolute concentration is an important feature on which local implementations of water regulation standards are based (Länderarbeitsgemeinschaft für Wasser (LAWA) 1998). Additionally, we aimed at detecting changes in the hydrochemistry including dilution processes. If the relative concentrations of cations and anions does not change, but the total concentration does, these would go unnoticed using normalized data.

We compared the results of both clustering methods and assessed the congruence between them by comparing how many data points are grouped in the same cluster by both clustering methods. Both methods were tested for their sensitivity by removing one of the eight ions at a time before running the clustering algorithm with the remaining data.

The resulting workflow groups yearly data points into a natural state before defining corridors which represent “within the natural fluctuation range”, and “outside of the natural fluctuation range” based on the mean and standard deviation (SD) of the cluster representing the natural state. Once the proposed workflow was checked to detect the outliers of distinct fluctuation events, we tested it for its suitability as an early warning system. To do so, we applied the workflow to a discernible fluctuation event in three iterations, increasing the data points available to the clustering algorithm with each iteration. We then assessed whether the proposed corridors would have allowed the distinction of the fluctuation event before and during its occurrence.

## Results

### General Hydrochemical Characteristics

**Fig. 2:**
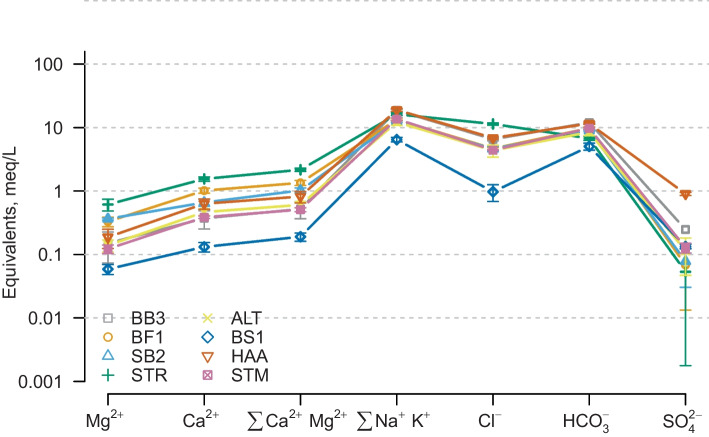
Schoeller Diagram of all eight assessed wells. Connected points are the arithmetic mean of all analyses at this well, error ars show one standard deviation

Figure 2 presents the hydrochemical composition of all 8 wells in an aggregated Schoeller diagram. Waters with identical characteristics but varying concentrations plot in parallel lines. The concentrations of the individual ions differ by one order of magnitude. While the general characteristics seem to be fairly similar, there are significant differences where lines cross and/or the slope of the lines deviates strongly from the general trends. Water from BS1 shows low TDS and a dominance of sodium over chloride, indicating ion exchange processes along the flow path and little contact to saline waters. In contrast, STR show high concentration values with sodium concentrations almost matching chloride concentrations. This suggests a contribution of saline waters and little ion exchange.

In general, SD is low except for sulfate, pointing towards analysis errors rather than changes in the reservoir. Most thermal waters contain reduced sulfur (HS-, H2S) in significant concentrations. These species can oxidize during sampling and sample transfer unless special treatment is applied (Mayrhofer et al. 2014).

STM, HAA and ALT produce sodium-bicarbonate-chloride thermal waters. Relative equivalent concentrations of chloride decrease from west to east. BS1 stands out with relative chloride concentrations of less than 20 % and very low TDS.

The hydrochemical characteristics show that the waters from DEGK1110 and GK100158 differ significantly. This suggests different lithostratigraphic settings and local flow paths to the wells, which might include contact with aquifers above and below the main flow path. Regional residence times are long compared to reaction kinetics in the carbonate matrix. Thus, local effects influence the hydrochemical stability more strongly than the regional flow regime.

### Inflow Path Types

**Fig. 3:**
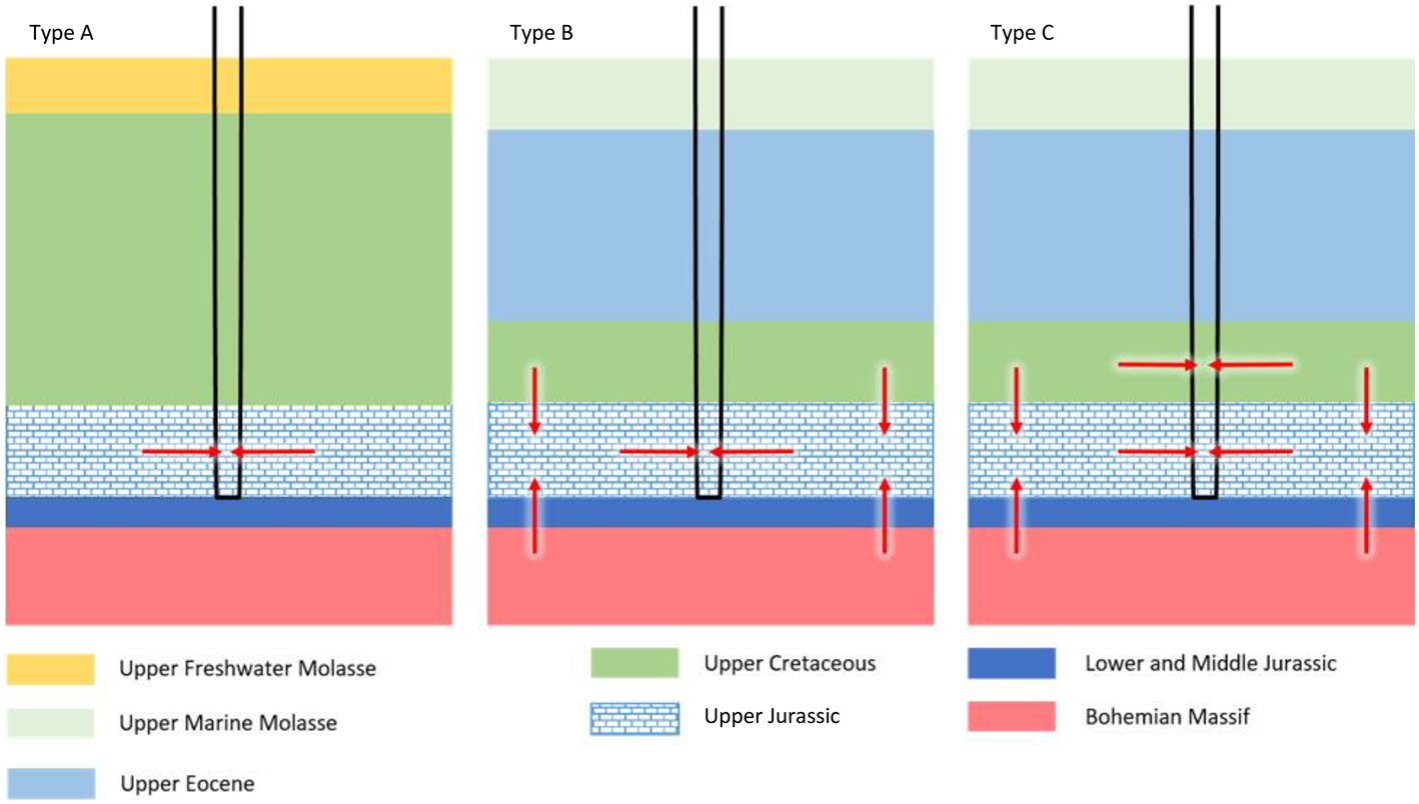
Inflow path types in the study area

Based on drilling logs (Baumann and Nießner 2012; Institut für Wasserchemie TUM 1999, Elster et al. 2016) and the hydrochemical characteristics, we propose three inflow path types (Fig. 3). These simplified types experience effects of residence times, extraction volumes and pressure regime in different ways. Type A aquifers are enclosed between impermeable layers. Regardless of the withdrawal rates and hydraulic potential, the water flows only in the host rock of the aquifer. In Type B aquifers, hydraulic contact with adjacent layers is possible. The magnitude of this influx is a function of the permeability of the main aquifer and its neighboring strata, and the hydraulic potential in each layer. Type C represents a technical connection of different aquifers in strata which are otherwise separated. Here, the amount of mixing from the different aquifers is a function of the transmissivity of the different layers, the hydraulic potential and the production rates. While this exploration strategy is deprecated, some wells of this type still exist. These three inflow types provide a quick method for a first assessment of the robustness of the wells’ exploitation. Out of all the wells in this study, 2 (SB2 and STR) belong to Type A, 4 (BF1, ALT, BS1, SB2) are Type B wells and 2 (BB3, HAA) are Type C wells. The majority of Type B wells are observed in the central part of the NAFB.

### Well Specific Characteristics

**Fig. 4:**
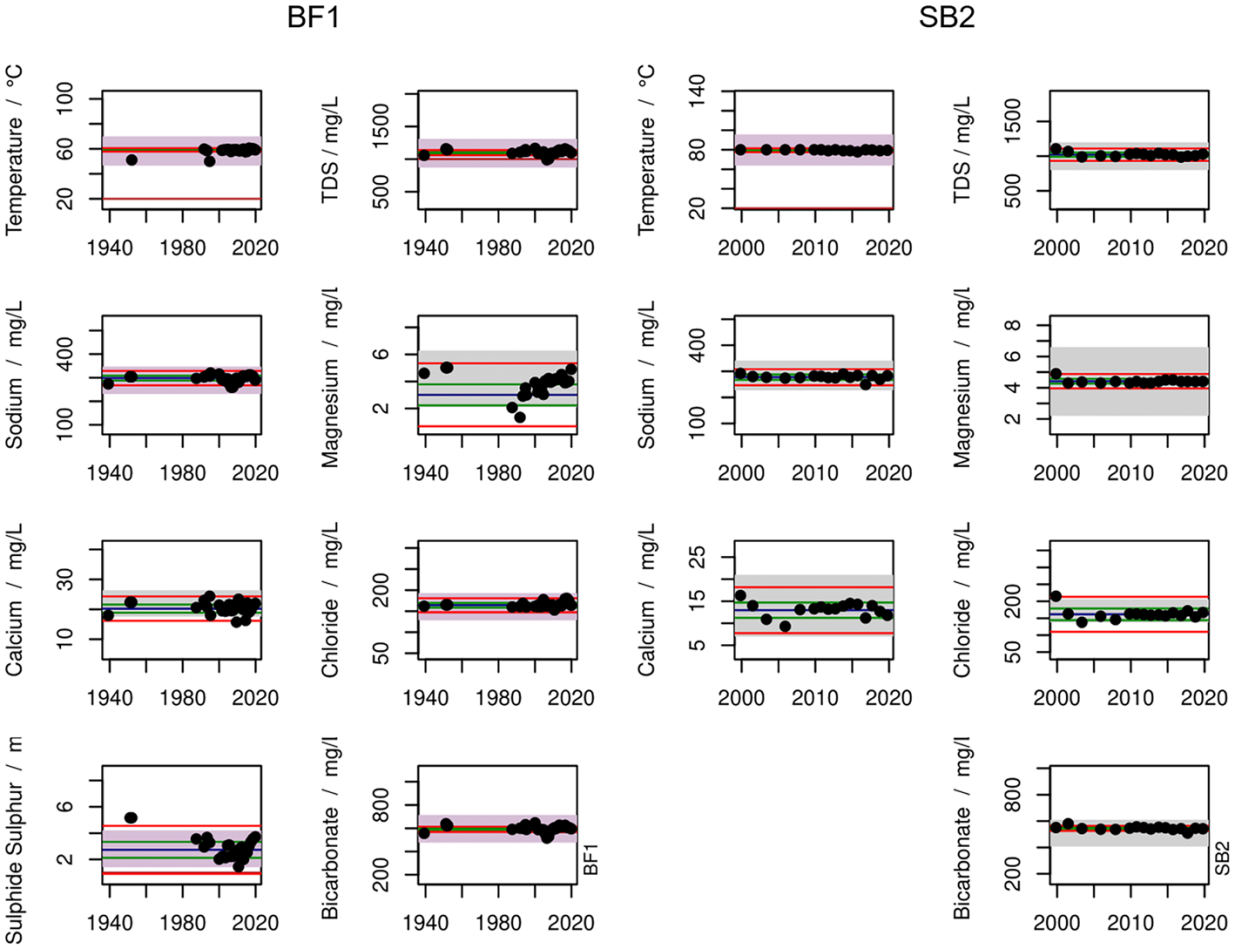
Time series of characteristic ions at BF1 and SB2. The boxes show the allowed ± 20 % variation intervals (± 50 % for parameters with a relative concentration below 20 %) according to the legal framework (grey boxes: parameters not relevant to the hydrochemical characterization). The lines show the reference value (last official analysis; blue), the NC (green), and the AC (red)

For the remainder of this study, we will focus on two wells: BF1 (Type B) and SB2 (Type A), covering both variability in inflow types and the robustness of hydrochemical conditions. BF1 is used for heating and balneological purposes. It belongs to a group of wells with a net discharge and no injection wells. SB2 exclusively generates heat for local district heating and is connected to an injection well to maintain the water balance.

On the left side of Fig. 4, selected ions and parameters for BF1 at all available sampling dates are shown. The temperature is constant without any discernible trend. The few low temperature data points were likely not measured at the well-head but elsewhere along the surface level production line.

Sodium and bicarbonate develop similarly over time. Between 1998 and 2011, sodium and bicarbonate display a highly dynamic behaviour, starting with an increase in concentration reaching maximum values of 296.5 mg/L and 646.8 mg/L, respectively. During the following years, the concentration values decrease until they reach minimum values of 260 mg/L and 515 mg/L, respectively, in 2006. After this development, sodium and bicarbonate values level off around a stable mean value with no apparent short-term trends.

Overall, BF1 is characterized by relatively large fluctuations in its chemical composition. This corresponds with its inflow path type (Type B). Here, the main inflow stems from the Upper Jurassic’s carbonates, with contributions from the overlying Coniac/Cenoman carbonate formations. Figure 4 suggests a similar hydrochemical character with slightly lower mineralization for these two aquifers.

Since BF1 ion concentrations lie mostly within allowed limits, one might certify a good status for the aquifer. However, the dynamic behaviour around 2006 is striking (Fig. 4). The threshold of 1 g/L TDS was almost undercut and the concentration of sulfide undercut the allowed fluctuation range once. This illustrates that the criteria set by the current legal framework are not sensitive enough to detect changes possibly indicating unsustainable well development.

On the right side, Fig. 4 depicts the same selection of parameters as above for all available sampling dates at SB2. Most parameters are constant without any trend. Although TDS occasionally drops below 1000 mg/L, this is negligible because the water is not used for balneological purposes.

In general, SB2 shows a stable development. TDS fluctuates only at the beginning of the recorded data and levels off at approximately 1000 mg/L. Calcium and chloride show the strongest variability, with their largest fluctuations occurring during the first ten years of the timeline. Their initial values are 16.3 mg/L and 215 mg/L, respectively. Within a decade, they decrease to minimum values of 9.3 mg/L in 2005 and 138 mg/L in 2003, before they settle on stable values at around 13 mg/L and 160 mg/L, respectively. Except for chloride, none of the parameters exceeds or undercuts the permissible fluctuation ranges.

SB2 generally presents a stable well representing its assigned inflow path type (Type A). Main inflow stems from the Upper Jurassic, which is shielded from the influence of waters from adjacent formations by impermeable layers. Thus, even increased production rates are unlikely to result in major changes in TDS or individual ion concentrations.

### A New Workflow for Defining Baseline Fluctuations

It is in the interest of well operators and authorities to establish an early warning system with a high sensitivity to changes in the the overall state of the aquifer, such as presented in Fig. 4. It is not within the scope of this study to determine whether these changes are caused by additional exploration activities, over-use, or long-term changes of the hydraulic regime. The focus here lies on detecting fluctuations before they become legally relevant (e.g. by undercutting a minimum TDS threshold of 1000 mg/L), which is why our proposed framework must function as an early warning system. We suggest a workflow which produces two corridors which shall be referred to as natural corridor (NC), representative for natural variations at sustainable use, and action corridor (AC) indicating unsustainable use, respectively. The latter should trigger further investigations and/or measures to retain a good state of the aquifer.

Both corridors are based on a specific well’s hydrochemical character, including, where applicable, trace ingredients relevant to balneotherapy. We assume that every well has a natural hydrochemical variance which reflects its lithostratigraphic setting and inflow type. This natural variance includes production from the well, as there are usually no prior data from the aquifer itself.

**Fig. 5:**
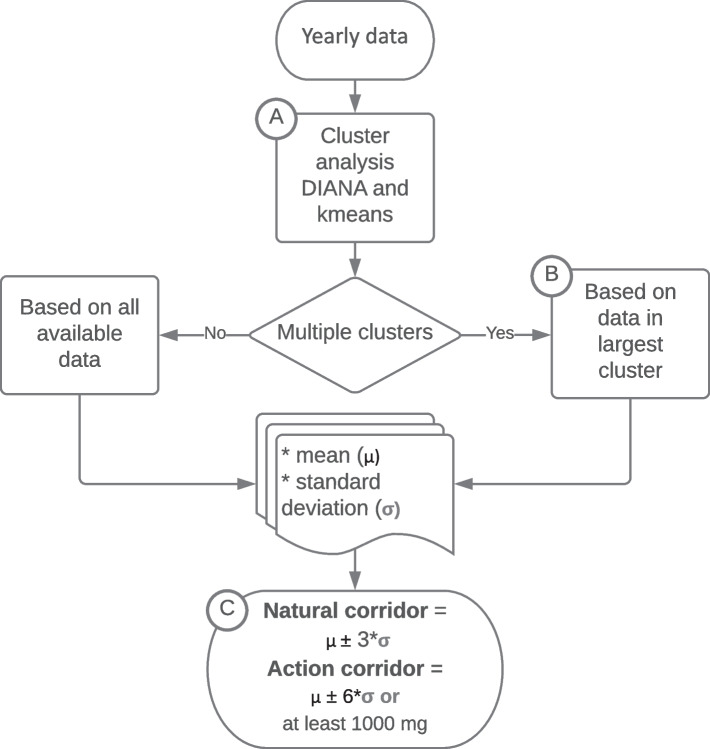
Clustering workflow identifying the natural fluctuation range and action corridor of a given well

The corridors are defined as 3 times (NC) and 6 times (AC) the SD around the Blank (mean values of the analyses representative for a natural state; Fig. 5c). This delineation of the corridors picks up the definitions of the limit of detection ($\bar{x}_{b} + 3 \sigma$) and the limit of quantification ($\bar{x}_{b} + 6 \sigma$) (Armbruster and Pry 2008). Here $\bar{x}_{b}$ is the mean of all samples representing the natural fluctuation range and $\sigma$ their SD. Following the three-sigma rule (or 68-95-99.7 rule) in probability theory (Hao et al. 2015) for a random variance with normal distributed values, 3 $\sigma$ cover 99.73 %, while 6 $\sigma$ cover 99.999998 % of the values.

This definition seems straightforward under the premise that the natural state of the well is known. If the hydrochemical signature of a given water is as constant as shown in Fig. 4, the natural state is evident. However, BF1 shows some features which apparently do not result from natural variation (Fig. 4). Therefore, the definition of the corridors requires the additional step of delineating the natural state first.

Using clustering algorithms (DIANA and k-means; Fig. 5a), we divide the data set into several groups of analyses. Historic data of all wells in the study area support the assumption that the natural state is represented by the largest resulting cluster (Fig. 5b).

Preliminary tests on whether there are any direct effects of the production rate on the hydrochemical characteristics are mandatory and can be obtained during pumping tests. Effects of the pumping rates and/or changes in the hydraulic potential on the hydrochemical composition are likely in inflow types B and C. Here, the concept of determining the natural variation is going to fail if the yearly samples are taken at different pumping rates. Table 1 and Fig. 4 display the results of the proposed workflow for all eight wells.

The mean of the NC and the mean of all data points available often lie close together. Differences arise in SD values which are typically much smaller for the NC than those of the entire data set. This underlines the importance of selecting the data points making up the well’s natural range in the attempt of defining its baseline fluctuation.

**Table 1:** Congruence of cluster assignment to each data point between DIANA and k-means and clustering results for DIANA

Well name	Available analyses	Sampled period	Congruence	Mean (NC)	Mean (total)	SD (NC)	SD (total)	n within NC
BB3	28	1973 - 2019	96	1457.99	1423.70	19.33	48.73	15
BF1	26	1939 - 2019	89	1106.10	1112.84	12.17	40.55	14
SB2	16	1999 - 2019	100	1024.80	1029.17	21.87	30.18	14
STR	26	1990 - 2019	77	1269.35	1236.55	39.55	50.79	14
ALT	11	1990 - 2010	64	1230.97	1236.55	7.48	50.79	10
BS1	5	1922 - 2003	100	527.77	502.15	27.40	40.15	3
HAA	5	1992 - 2009	100	1457.80	1432.14	48.47	71.09	4
STM	9	1999 - 2011	100	1096.97	1093.28	11.04	24.82	5

Table 1 further shows the congruence of DIANA and k-means. K-means was run with $n=3$ classes. Generally high congruence values are observed. ALT shows the lowest congruence value (64 %). For this data set, the k-means cluster analysis resulted in two equally large clusters. Increasing the number of clusters from three to four classes for both algorithms would increase the congruence value for ALT to 100 %. Excluding ALT, the minimum congruence value for all other wells is 77 % and the average congruence value, excluding ALT, is 94.57 %.

The results of the clustering were robust to the removal of most single ions from the data (Online Resources 3 and 4). The NC did not change when excluding ${Ca^2+}, {K+}, {Mg^2+}, {F-}$, and ${SO4^2-}$. Excluding Na+ or HCO3- did change the results because these ions are the main constituents of the water at these wells. K-means was sensitive to the removal of Cl- at BF1, which is attributed to the predefined number of clusters in k-means (SI). The two clustering algorithms are in accordance regarding the definition of the NC, and the absence of Na+ affects both clustering algorithms in the same way. In SB2, the number of data points in the largest cluster did not change when using DIANA. Using k-means, it changed from 16 to 11 when excluding Cl-, and to 10 when excluding HCO3-. Using k-means on BF1, the numbers of data points in the largest cluster increased from 14 to 16 when Cl- was excluded, and to 19 when Na+ was excluded. When using DIANA on BF1, these numbers changed from 14 to 19 when excluding Na+, and to 21 when excluding HCO3-.

### Workflow Application

Figure 4 shows the resulting corridors for the two wells BF1 and SB2 based on DIANA clustering. Since 1939, TDS at BF1 was outside the NC with ten analyses, two data points are even outside the AC. For SB2, only one TDS data point is outside the NC and no data points cut the AC. With a TDS of 1112.8 ± 40.6 mg/L, BF1 generally reveals a larger variance compared to SB2 (TDS = 1029.17 ± 30.2 mg/L). However, their respective largest clusters reveal a different statistical signature: BF1’s largest cluster exhibits a TDS of 1106.1 ± 12.2 mg/L, while SB2’s largest cluster has a TDS of 1024.8 ± 21.9 mg/L. In BF1, the values attributed to the NC cluster lie much closer together. In SB2, there are only two values different enough from the other data points to form their own clusters. Thus, the entire remainder of the data set is grouped into one cluster, resulting in a larger NC for SB2. Our cluster analysis-based corridors are much narrower than the state of the art ± 20 % corridors around the last measurement. On the other hand, our corridors for single parameters, such as magnesium in BF1, might be more lenient than the ± 50 % corridors. This is because instead of relying on a single value, our corridors take into account the entire time series, providing a more robust assessment.

In order to address whether this workflow is apt to function as an early warning system, we assessed the development of corridors with increasing amounts of available data points for BF1 (SI), beginning with five data points covering the period from 1987 to 1995 (Online Resource 4). The DIANA clustering algorithm was chosen for this purpose because it allows insight into the development of the clustering structure through its visualization of dendrograms. The largest cluster contains four out of these five initial data points. By adding five more data points leading up to the fluctuation event (distinct and sudden decrease in total mineralization values in the early 2000s), the new thresholds based on now ten data points delineate slightly narrower corridors due to a lower SD in the newly formed largest cluster (Online Resource 4). After ten measurements we observe two data points exceeding the NC in 1994 and 2000. During the entire iteration, varying data points are clustered in the natural range, which explains the changes in SD and the NCs. When running the clustering workflow on 14 measurements, the data set covers the entire aforementioned sudden dip in TDS values observed between 2005 and 2007. The widths of the corridors change again very slightly, based on a new set of analysis data now making up the largest cluster. While the largest cluster’s SD changes noticeably, the respective mean values, representing the well’s range of TDS, stay relatively constant throughout the three scenarios. Both corridors based on the first and last scenarios would have managed to detect this fluctuation event with their respective thresholds, proving the applicability of the proposed workflow for early warning purposes.

## Discussion

The Upper Jurassic deep geothermal aquifer in the NAFB is an important source of geothermal water. Multiple well operators exploit it for both balneological and geothermal purposes. Even though this groundwater constitutes a non-renewable resource, the WFD is not an adequate tool to protect the Upper Jurassic aquifer, since the directive was designed to be used on shallow aquifers. Local implementations to monitor an aquifer’s water quality (Deutscher Heilbäderverband and Deutscher Tourismusverband 2016; Daughney and Reeves 2006) are faced with the major problem of their application being based on arbitrary and generic thresholds in order to indicate problematic exploitation procedures. Daughney and Reeves (2006) stress the importance of defining the baseline fluctuation range in order to determine unnatural developments. Despite its easy and straight forward approach, the *Begriffsbestimmungen* (Deutscher Heilbäderverband and Deutscher Tourismusverband 2016) fail to delineate a given well’s specific inherent and natural hydrochemical fluctuation range. Further, it offers no quantitative analysis of sudden changes in the hydrochemical signature as long as these, sometimes even distinct, events do not cross the aforementioned generic thresholds. Thus, significant yet not large enough fluctuations, which might indicate an unsustainable use of a well, remain unnoticed.

This study offers a new statistical approach to define a well’s natural state. We propose a framework which, while slightly more time-consuming and complex than applying a generic percentage to a single data point, offers various advantages. By using clustering analysis, we found that we can robustly identify the specific natural fluctuation range of a well’s hydrochemical signature, and detect changes in a well’s hydrochemical composition which are not part of the natural fluctuation range. We identify these as data points that leave the previously defined NC. By applying well-specific corridors, it is no longer necessary for these unnatural events to exceed a threshold disconnected from the inherent natural fluctuation range of a well.

We used two clustering methods (DIANA and k-means). The k-means algorithm is relatively simple to implement and produces results that are easy to interpret. However, one has to have good knowledge of the data in order to choose the right value for *k*, a parameter on which the entire clustering mechanism then depends. Further, k-means tends to exhibit problems with defining clusters of varying densities (Likas et al. 2003) and the resulting clusters can be dragged by outliers (Wagstaff et al. 2001). DIANA, while being more complex than agglomerative clustering, does not require the user to define any initial parameters. Divisive algorithms take into account the global data distribution at the beginning of the clustering process, making them more accurate than agglomerative algorithms. A value to define the clusters, namely the height value from which the clusters are derived, must be defined by the user. We found that DIANA and k-means had a high congruence in the resulting cluster structures, which suggests that both algorithms are adequate tools to define the set of analyses representative for the well’s naturals state. Four out of eight examined wells show a congruence of 100 %, and the lowest congruence is 64 %. Even this number can be improved once the number of clusters (*k*) is adjusted in the k-means clustering step. When assessing the robustness of the approaches regarding the absence of individual parameters, small changes in the resulting corridor widths were observed. The largest discrepancies occurred when leaving out HCO3-. Here, k-means includes a large number of analyses values in the NC which seem to be part of fluctuation events. While both algorithms handle the data sets well, DIANA seems to be more robust compared to k-means. This is attributed to the fixed number of clusters in k-means. Although the clustering was robust to the exclusion of single ions, this only shows that the assessed wells react to stress with a change in multiple hydrochemical parameters. Trace metals, polycyclic aromatic hydrocarbons and isotopes could constitute additional parameters to detect changes in the overall flow pattern, however, too few analyses that included these parameters were available (regular intervals for the extended analyses were 10 years and are now 5 years). As the total concentration of the ions is a feature of the hydrochemical composition, clustering on non-normalized data is preferred.

The sensitivity of the newly developed framework to detect changes in the flow regime to the wells was tested on the well BF1. This well exhibits a clear fluctuation event which previously went unnoticed using the generic approach described by the GSA (Deutscher Heilbäderverband and Deutscher Tourismusverband 2016). In contrast, the new workflow was able to establish an NC and AC which would have detected the fluctuation events based on just five prior yearly data points. This is significant because it shows that the event was not only discernible retrospectively, but that the proposed workflow would have detected it by the time it occurred. It is important to consider that each new data point hones the precision of the clustering workflow and may thus change the well’s natural fluctuation range slightly. This was observed when testing the workflow with differing amounts of data points at the same well. Nevertheless, this test produced significant conclusions regarding the minimum sample size of a data set which is to be used for this framework. According to our findings, a minimum of 5 yearly data points may offer a good base to establish a well’s natural fluctuation range. Regarding data quality, a certain variance and offset has to be expected due to updated sampling and analysis protocols, as well as improved analytical methods, when using older measurements, and/or from different laboratories. This must be considered in the quality of the clustering structure.

## Conclusion

This paper set out to design a robust statistical workflow by which the natural state of an individual well’s hydrochemical signature can be defined and unsustainable operation strategies determined and avoided. A major goal was to discern unnatural fluctuation events, which might hint towards unsustainable well development before it becomes legally problematic for the operators. We propose a cluster analysis-based workflow using agglomerative and divisive algorithms as a substitute for the state-of-the art generic approach of arbitrarily-set thresholds for allowed minimum and maximum concentrations of certain hydrochemical parameters. Our framework is a practical approach to address the conflict of intensive geothermal water extraction and deep groundwater being a limited resource, and offers the following key features:

1. The proposed cluster analysis-based workflow offers well-specific identification of the natural hydrochemical fluctuation range focusing on total mineralization (sum of eight main ions and additional characterizing trace ions). In addition to the natural fluctuation range, it determines two corridors, delineated by a natural threshold and an action threshold. These thresholds can be utilized by surveyors tasked with assessing the sustainability of a well’s operation procedures.
2. The proposed framework proved to be successful in detecting unnatural fluctuation events that would have gone unnoticed by the state-of-the-art approach of setting generic minimum and maximum concentration values. It is thus sensitive to changes in a well’s hydrochemical signature while at the same time considering its natural fluctuation regime. Every newly added data point hones the accuracy of the determined natural fluctuation. This is particularly important because deep geothermal data is notoriously scarce and difficult to sample on high spatial and temporal resolutions.
3. The presented framework is suitable for the application as an early warning system. Based on the case study of a well whose waters exhibited a strong fluctuation event, the corridors produced by our workflow would have been able to detect the fluctuation before and while it occurred. This assessment also showed that the minimum data set size for this workflow is 5 yearly data points.

Finally, the results of the proposed workflow indicate that the wells exploiting the deep groundwater aquifers DEGK1110 and GK100158 are robust and previous exploration activities have not led to changes in the general state of the aquifers.

## Supplementary Information

Below is the link to the electronic supplementary material.

Online Resource 1 (PDF 1.76 MB)

## Data Availability

Hydrochemical data from Bavarian and Austrian wells were provided through the Technical University of Munich and/or upon request by the respective well operators.

## Code Availability

All code was written using the statistical software R. The software as well as the relevant clustering packages can be downloaded under https://www.r-project.org/.

## References

[CR1] Arle J, Bartel H, Baumgarten C et al (2017) Wasserwirtschaft in Deutschland. Grundlagen, Belastungen, Maßnahmen. Tech Rep Umwelt Bundesamt, Dessau-Roßlau. www.umweltbundesamt.de/publikationen

[CR2] Armbruster D, Pry T (2008) Limit of blank, limit of detection and limit of quantitation. Clin Biochem Rev 29 Suppl:19–52. https://pubmed.ncbi.nlm.nih.gov/18852857/PMC2556583 18852857

[CR3] Auguie B, Antonov A (2017) gridExtra: Miscellaneaous functions for "Grid" Graphics. https://cran.r-project.org/web/packages/gridExtra/index.html

[CR4] Baiocchi A, Lotti F, Piscopo V (2013) Impact des prélèvements d’eau souterraine sur l’interaction avec un complexe d’aquifères dans la zone géothermique de Viterbo (Centre de l’Italie). Hydrogeol J 21(6):1339–1353. 10.1007/s10040-013-1000-5

[CR5] Baumann T (2013) Organische Spurenstoffe in Tiefengrundwasserleitern. Jh Ges Naturkde Sonderband Carlé pp. 103–117

[CR6] Baumann T, Nießner R (2012) Bad Füssing Therme I Heilquellenanalyse, unveröffentlichtes Gutachten (Institut für Wasserchemie) (unpublished)

[CR7] Birner J, Fritzer T, Jodocy M et al (2012) Hydraulische Eigenschaften des Malmaquifers im Süddeutschen Molassebecken und ihre Bedeutung für die geothermische Erschließung. [Hydraulic characterisation of the Malm aquifer in the South German Molasse basin and its impact on geothermal exploitations]. Z Geol Wiss 40(2/3):133–156. http://zgw-online.de/en/media/133-122.pdf

[CR8] Daughney CJ, Reeves RR (2006) Analysis of temporal trends in New Zealand’s groundwater quality based on data from the National Groundwater Monitoring Programme. J Hydrol New Zeal 45(1):41–62. https://www.jstor.org/stable/43944938

[CR9] Deutscher Heilbäderverband, Deutscher Tourismusverband (2016) Begriffsbestimmungen / Qualitätsstandards für Heilbäder und Kurorte, Luftkurorte, Erholungsorte - einschließlich der Prädikatisierungsvoraussetzungen - sowie für Heilbrunnen und Heilquellen. Tech Rep Deutscher Tourismusverband e.V. und Deutscher Heilbäderverband e.V

[CR10] Elster D, Goldbrunner J, Wessely G et al (2016) Erläuterungen zur geologischen Themenkarte Thermalwässer in Österreich 1:500 000. Geologische Bundesanstalt

[CR11] European Parliament and Council (2000) Directive 2000/60/EC I - The European Water Framework Directive

[CR12] Ferguson G, Cuthbert MO, Befus K et al (2020) Rethinking groundwater age. Nat Geosci 13(9):592–594

[CR13] Foster S, Custodio E (2019) Groundwater Resources and Intensive Agriculture in Europe - Can Regulatory Agencies Cope with the Threat to Sustainability? Water Resour Manag 33(6):2139–2151. 10.1007/s11269-019-02235-6

[CR14] Fu KS, Cover TM, Diday E et al (1976) Digital Pattern Recognition. Springer, Berlin Heidelberg

[CR15] Goldscheider N (2005) Karst groundwater vulnerability mapping: Application of a new method in the Swabian Alb. Germany. Hydrogeol J 13(4):555–564. 10.1007/s10040-003-0291-3

[CR16] Hao Y, Cao H, Qi Y, et al (2015) Efficient keyword search on graphs using MapReduce. Proc - 2015 IEEE Int Conf Big Data pp 2871–2873. 10.1109/BigData.2015.7364106

[CR17] Heine F, Zosseder K, Einsiedl F (2021) Hydrochemical Zoning and Chemical Evolution of the Deep Upper Jurassic Thermal Groundwater Reservoir Using Water Chemical and Environmental Isotope Data. Water 13:1162. 10.3390/w13091162

[CR18] Hussain M, Ahmed SM, Abderrahman W (2008) Cluster analysis and quality assessment of logged water at an irrigation project, eastern Saudi Arabia. J Environ Manage 86(1):297–307. 10.1016/j.jenvman.2006.12.007 17590259 10.1016/j.jenvman.2006.12.007

[CR19] Institut für Wasserchemie TUM (1999) Begutachtung der Thermalbohrung 1 in Straubing (unpublished). Technical University Munich, Tech. rep

[CR20] Institut für Wasserchemie TUM (1999) Begutachtung des Wassers der “Chrysanti-Quelle” in Bad Birnbach (unpublished). Technical University Munich, Tech Rep

[CR21] Kang M, Ayars JE, Jackson RB (2019) Deep groundwater quality in the southwestern United States. Environ Res Lett 14(3). 10.1088/1748-9326/aae93c

[CR22] Kassambara A, Mundt F (2020) factoextra: Extract and Visualize the Results of Multivariate Data Analyses. http://www.sthda.com/english/rpkgs/factoextra

[CR23] Kaufman L, Rousseeuw P (1990) Finding Groups in Data: An Introduction to Cluster Analysis. Wiley, Brussels, Brussels

[CR24] Kim JH, Yum BW, Kim RH et al (2003) Application of cluster analysis for the hydrogeochemical factors of saline groundwater in Kimje. Korea. Geosci J 7(4):313–322. 10.1007/bf02919561

[CR25] Kodinariya TM, Makwana DPR (2016) Review on determining of cluster in K-means clustering review on determining number of cluster in K-means clustering. Int J 1(July):90–95. www.ijarcsms.com

[CR26] Länderarbeitsgemeinschaft für Wasser (LAWA) (1998) Richtlinien für Heilquellenschutzgebiete

[CR27] Lee RCT (1981) Clustering analysis and its applications. In: Tou JT (ed) Adv Inf Syst Sci Springer, Boston, Chap 4 p. 169–292

[CR28] Li F, Feng P, Zhang W et al (2013) An Integrated Groundwater Management Mode Based on Control Indexes of Groundwater Quantity and Level. Water Resour Manag 27(9):3273–3292. 10.1007/s11269-013-0346-8

[CR29] Likas A, Vlassis N, J. Verbeek J (2003) The global k-means clustering algorithm. Pattern Recognit 36(2):451–461. 10.1016/S0031-3203(02)00060-2

[CR30] Maechler M, Rousseeuw P, Struyf A, et al (2021) cluster: Cluster Analysis Basics and Extensions. R package version 2.1.2. https://cran.r-project.org/package=cluster

[CR31] Mayrhofer C, Niessner R, Baumann T (2014) Hydrochemistry and hydrogen sulfide generating processes in the Malm aquifer, Bavarian Molasse Basin. Germany. Hydrogeol J 22(1):151–162. 10.1007/s10040-013-1064-2

[CR32] Pacheco Castro R, Pacheco Ávila J, Ye M et al (2018) Groundwater Quality: Analysis of Its Temporal and Spatial Variability in a Karst Aquifer. Groundwater 56(1):62–72. 10.1111/gwat.12546 10.1111/gwat.12546 28618449

[CR33] Panagos P, Van Liedekerke M, Yigini Y et al (2013) Estimating soil organic carbon in Europe based on data collected through an European network. J Environ Public Health 24:439–450. 10.1016/j.ecolind.2012.07.020 10.1155/2013/158764 PMC3697397 23843802

[CR34] Patnaik AK, Bhuyan PK, Krishna Rao KV (2016) Divisive Analysis (DIANA) of hierarchical clustering and GPS data for level of service criteria of urban streets. Alexandria Eng J 55(1):407–418. 10.1016/j.aej.2015.11.003

[CR35] PostGIS Project Steering Committee (PSC) (2021) PostGIS v. 3.1.4. https://www.postgis.net

[CR36] PostgreSQL Global Development Group (2021) PostgreSQL Database System v. 13.5. http://www.postgresql.org/about/

[CR37] QGIS Development Team (2021) QGIS Geographic Information System v. 3.22. https://www.qgis.org

[CR38] R Core Team (2020) R: a language and environment for statistical computing. https://www.r-project.org/

[CR39] Ribeiro L, Macedo ME (1995) Application of multivariate statistics, trend- and cluster analysis to groundwater quality in the Tejo and Sado aquifer. Groundw Qual Remediat Prot Proc Conf Prague, 1995 January(95):39–47. https://www.researchgate.net/profile/Luis-Ribeiro-33/publication/252068832_Application_of_multivariate_statistics_trend-_and_cluster_analysis_to_groundwater_quality_in_the_Tejo_and_Sado_aquifer/links/0f31753454ae3ace43000000/Application-of-multivariate-st

[CR40] Sayemuzzaman M, Ye M, Zhang F et al (2018) Multivariate statistical and trend analyses of surface water quality in the central Indian river Lagoon area. Florida. Environ Earth Sci 77(4):1–13. 10.1007/s12665-018-7266-0

[CR41] Thyne G, Güler C, Poeter E (2004) Sequential analysis of hydrochemical data for watershed characterization. Ground Water 42(5):711–723. 10.1111/j.1745-6584.2004.tb02725.x 15457794 10.1111/j.1745-6584.2004.tb02725.x

[CR42] Ungemach P, Antics M, Papachristou M (2005) Sustainable Geothermal Reservoir Management. Proc World Geotherm Congr April(05):24–29. https://d1wqtxts1xzle7.cloudfront.net/43589480/Sustainable_Geothermal_Reservoir_Managem20160310-10466-hkii8l-with-cover-page-v2.pdf?Expires=1646221751 &Signature=BwyQ2XXEKN8GyolI1lBwiY5uI5qprAbEZOo1du8t6GW9LIJjiN61T0QpIqYr4npR8oS3swDgvp34YHivm19jDKNxYmo-yO

[CR43] Wagstaff K, Cardie C, Rogers S, et al (2001) Constrained K-means Clustering with Background Knowledge. Int Conf Mach Learn ICML pages:577–584. http://citeseerx.ist.psu.edu/viewdoc/download?doi=10.1.1.90.4624 &rep=rep1 &type=pdf

[CR44] Wang H, Jiang XW, Wan L et al (2015) Hydrogeochemical characterization of groundwater flow systems in the discharge area of a river basin. J Hydrol 527:433–441. 10.1016/j.jhydrol.2015.04.063

[CR45] Wycisk P, Weiss H, Kaschl A et al (2003) Groundwater pollution and remediation options for multi-source contaminated aquifers (Bitterfeld/Wolfen, Germany). Toxicol Lett 140–141:343–351. 10.1016/S0378-4274(03)00031-6 10.1016/s0378-4274(03)00031-6 12676483

[CR46] Yang J, Ye M, Tang Z, et al (2020) Using cluster analysis for understanding spatial and temporal patterns and controlling factors of groundwater geochemistry in a regional aquifer. J Hydrol 583(January). 10.1016/j.jhydrol.2020.124594

