# Supplementary material for: Assessing Sustainable Development of Deep Aquifers
Source: Water Resour Manag (Dordr). 2023 May 16:1–18. Online ahead of print. doi: 10.1007/s11269-023-03529-6 (PMC10186284; doi:10.1007/s11269-023-03529-6)
Supplement: Supplementary file 1 — Online Resource 1 (PDF 1.76 MB) [file 11269_2023_3529_MOESM1_ESM.pdf]

Information

Springer Nature 2021 LaTeX template

Click here to access/download;attachment to manuscript;Supplementary_Information.pdf

Click here to view linked References

Assessing sustainable development of deep aquifers – Supplementary Information

Annette Dietmaier[1] and Thomas Baumann[1*]

[1*]TUM School of Engineering and Design, Chair of Geohydrology, Technical University Munich, Arcisstr. 21, Munich, 80333, Bavaria, Germany.

*Corresponding author(s). E-mail(s): tbaumann@tum.de; Contributing authors: annette.dietmaier@tum.de;

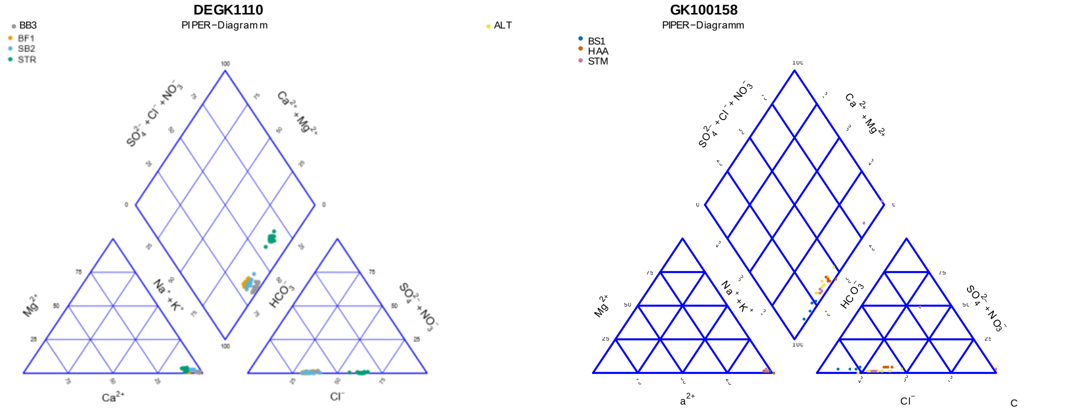

Fig. 1 Piper diagrams showing general hydrochemical composition of the assessed groundwater samples. Top graph shows wells in the GK100158 while those within DEGK1110 are displayed in the bottom graph.

Table 1 Results of mean and RSD values for TDS and each parameter when individual ions (Cl^-, HCO_3^- and Na^+) were left out in the clustering process in BF1. The top values are calculated by Diana, the bottom values are calculated by kmeans.

		All data		w/o Cl^-		w/o HCO_3^-		w/o Na^+	
		Mean /mg/L	RSD /%	Mean /mg/L	RSD /%	Mean /mg/L	RSD /%	Mean /mg/L	RSD /%
Na^+	Diana	295.65	0.03	295.65	0.03	301.26	0.02	297.87	0.03
	kmeans	295.65	0.03	296.31	0.03	308.01	0.02	297.87	0.03
K^+		19.84	0.21	19.84	0.21	17.75	0.13	19.31	0.19
		19.84	0.21	19.74	0.20	17.63	0.13	19.31	0.19
Ca^{2+}		21.11	0.15	21.11	0.15	21.18	0.13	20.89	0.14
		21.11	0.15	21.20	0.14	20.87	0.10	20.89	0.14
Mg^{2+}		3.52	0.30	3.52	0.30	3.87	0.25	3.67	0.26
		3.52	0.30	3.57	0.28	3.85	0.25	3.67	0.26
F^-		6.52	0.08	6.52	0.08	6.00	0.12	6.31	0.09
		6.52	0.08	6.48	0.08	5.68	0.18	6.31	0.09
Cl^-		162.57	0.04	162.57	0.04	166.39	0.04	164.45	0.04
		162.57	0.04	163.61	0.04	167.13	0.04	164.45	0.04
SO_4^{2-}		3.41	0.47	3.41	0.47	3.88	0.41	3.82	0.45
		3.41	0.47	3.52	0.44	4.39	0.31	3.82	0.45
HCO_3^-		593.47	0.01	593.47	0.01	606.85	0.02	597.52	0.02
		593.47	0.01	595.29	0.01	615.96	0.02	597.52	0.02
sum		1106.09	0.01	1106.09	0.01	1127.19	0.02	1113.84	0.02
		1106.09	0.01	1109.71	0.01	1143.53	0.01	1113.84	0.02

Table 2 Results of mean and RSD values for TDS and each parameter when individual ions (Cl^- and HCO_3^-) were left out in the clustering process in SB2. In contrast to BF1, the cluster structure did not change when excluding Na^+, which is why the corresponding column is not shown in Table 2. The top values are calculated by Diana, the bottom values are calculated by kmeans.

		All data		w/o Cl^-		w/o HCO_3^-	
		Mean /mg/L	RSD /%	Mean /mg/L	RSD /%	Mean /mg/L	RSD /%
Na^+	Diana	276.64	0.03	277.50	0.04	279.14	0.02
	kmeans	276.64	0.03	274.73	0.03	281.20	0.02
K^+		15.92	0.05	16.42	0.13	15.80	0.04
		15.92	0.05	15.90	0.05	15.62	0.03
Ca^{2+}		12.80	0.12	12.96	0.13	13.00	0.11
		12.80	0.12	12.51	0.12	13.60	0.06
Mg^{2+}		4.38	0.02	4.42	0.03	4.38	0.02
		4.38	0.02	4.38	0.02	4.38	0.02
F^-		5.81	0.05	5.87	0.06	5.77	0.05
		5.81	0.05	5.80	0.05	5.72	0.05
Cl^-		157.93	0.05	161.64	0.11	158.93	0.05
		157.93	0.05	156.82	0.05	163.10	0.03
SO_4^{2-}		4.16	0.47	3.62	0.31	4.16	0.47
		4.16	0.47	3.77	0.32	4.13	0.54
HCO_3^-		547.15	0.02	545.08	0.01	544.77	0.03
		547.15	0.02	542.75	0.01	546.30	0.03
sum		1024.80	0.02	1027.52	0.03	1025.94	0.02
		1024.80	0.02	1016.65	0.01	1034.05	0.02

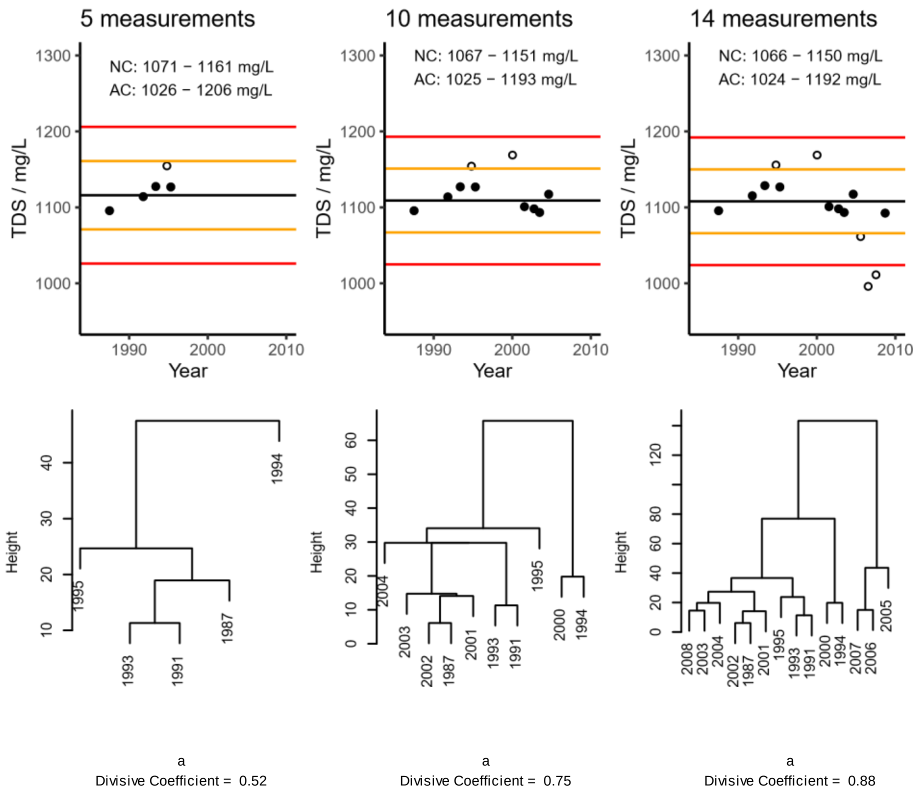

Fig. 2 Development of corridors with growing data set based on the Diana algorithm. The solid black dots represent the data points that were included in the main cluster.

Differences to previous submission Springer Nature 2021 LaTeX template Click here to access/download;attachment to manuscript;WARM-D-22-00461-diff.pdf

Click here to view linked References
